# Are sesquiterpene lactones the elusive KARRIKIN-INSENSITIVE2 ligand?

**DOI:** 10.1007/s00425-021-03571-x

**Published:** 2021-02-01

**Authors:** Mehran Rahimi, Harro Bouwmeester

**Affiliations:** Plant Hormone Biology Group, Green Life Sciences Cluster, Swammerdam Institute for Life Science, University of Amsterdam, Science Park 904, 1098 XH Amsterdam, The Netherlands

**Keywords:** 3D structure models, 8-Epixanthatin, Germination, Hypocotyl elongation, KAI2 signaling, Strigolactone

## Abstract

**Main conclusion:**

The sunflower sesquiterpene lactones 8-epixanthatin and tomentosin can bind to the hydrophobic pocket of sunflower KAI2 with an affinity much higher than for the exogenous ligand KAR.

**Abstract:**

Sesquiterpene lactones (STLs) are secondary plant metabolites with a wide range of biological, such as anti-microbial, activities. Intriguingly, the STLs have also been implicated in plant development: in several Asteraceae, STL levels correlate with the photo-inhibition of hypocotyl elongation. Although this effect was suggested to be due to auxin transport inhibition, there is no structural–functional evidence for this claim. Intriguingly, the light-induced inhibition of hypocotyl elongation in Arabidopsis has been ascribed to HYPOSENSITIVE TO LIGHT/KARRIKIN-INSENSITIVE2 (HTL/KAI2) signaling. KAI2 was discovered because of its affinity to the smoke-derived karrikin (KAR), though it is generally assumed that KAI2 has another, endogenous but so far elusive, ligand rather than the exogenous KARs. Here, we postulate that the effect of STLs on hypocotyl elongation is mediated through KAI2 signaling. To support this hypothesis, we have generated homology models of the sunflower KAI2s (HaKAI2s) and used them for molecular docking studies with STLs. Our results show that particularly two sunflower STLs, 8-epixanthatin and tomentosin, can bind to the hydrophobic pockets of HaKAI2s with high affinity. Our results are in line with a recent study, showing that these two STLs accumulate in the light-exposed hypocotyls of sunflower. This finding sheds light on the effect of STLs in hypocotyl elongation that has been reported for many decades but without conclusive insight in the elusive mechanism underlying this effect.

Sesquiterpene lactones (STLs) are secondary plant metabolites with a wide range of biological, such as anti-microbial, activities (Spring and Hager 1982; Chadwick et al. 2013; Liu et al. 2018). Intriguingly, the STLs have also been implicated in plant development: particularly in sunflower, STL levels correlate with the photo-inhibition of hypocotyl elongation (Spring and Hager 1982; Yokotani-Tomita et al. 1999; Arai et al. 2013). Although this effect was suggested to be due to auxin transport inhibition, there is no structural–functional evidence for this claim (Arai et al. 2013; Ueda et al. 2013). Intriguingly, the light-induced inhibition of hypocotyl elongation in Arabidopsis has been ascribed to HYPOSENSITIVE TO LIGHT/KARRIKIN-INSENSITIVE2 (HTL/KAI2) signaling (Sun and Ni 2011; Waters and Smith 2013). KAI2 was discovered because of its affinity to the smoke-derived karrikin (KAR), though it is generally assumed that KAI2 has another, endogenous but so far elusive, ligand rather than the exogenous KARs (Conn and Nelson 2016). Here, we postulate that the effect of STLs on hypocotyl elongation is mediated through KAI2 signaling. To support this hypothesis, we have generated homology models of the sunflower KAI2s (HaKAI2s) and used them for molecular docking studies with STLs. Our results show that particularly two sunflower STLs, 8-epixanthatin and tomentosin, can bind to the hydrophobic pockets of HaKAI2s with high affinity. Our results are in line with a recent study, showing that these two STLs accumulate in the light-exposed hypocotyls of sunflower (Spring et al. 2020). This finding sheds light on the effect of STLs in hypocotyl elongation that has been reported for many decades but without conclusive insight in the elusive mechanism underlying this effect.

A number of studies showed that in sunflower, exposure to light results in an increase in the concentration of STLs in the hypocotyl, which correlates with the inhibition of elongation (Spring and Hager 1982; Yokotani-Tomita et al. 1999; Arai et al. 2013; Ueda et al. 2013). Moreover, accumulation of the STL, 8-epixanthatin, in the blue light-exposed side of sunflower hypocotyls was demonstrated and suggested to inhibit elongation, thus causing curvature toward the light. And finally, the application of exogenous STLs such as 8-epixanthatin significantly reduces the elongation of hypocotyls (Yokotani-Tomita et al. 1999).

The inhibition of hypocotyl elongation under light is also a well-known characteristic of KAR perception by plants (Sun and Ni 2011; Waters and Smith 2013). KARs are compounds derived from smoke that inhibit the hypocotyl elongation of *Arabidopsis thaliana* under blue and red light. KARs bind to KAI2, and hence induce seed germination and inhibit hypocotyl elongation (Nelson et al. 2010; Waters and Smith 2013). There is thus a striking similarity between the hypocotyl elongation inhibitory effects reported for STLs and KARs. Studies on the Arabidopsis *kai2-2* mutant—that has an increased hypocotyl length—have suggested that KAI2 has an unknown endogenous ligand, coined KAI2 ligand (KL) (Conn and Nelson 2016). Most likely, KL acts in a similar fashion as KARs to activate KAI2 signaling, resulting in the same phenotypes. It is important to note that all efforts to identify KL have so far failed.

Over the past few years, a number of studies on the root parasitic Orobanchaceae showed that they have multiple copies of *KAI2/HTLs*. In *Striga hermonthica*, for example, the most studied parasitic plant species in this family, *KAI2* underwent extensive gene duplication and it has at least 11 *HTLs*. Intriguingly, these receptors were shown to perceive exogenous signals, the so-called germination stimulants that are exuded from the roots of their host plant, which ensures that germination occurs near a host root (Conn et al. 2015; Toh et al. 2015; Bouwmeester et al. 2020). The germination stimulants for most parasitic plant species belong to the strigolactones, an intriguing class of compounds with endogenous, hormonal, roles as well as rhizosphere signaling activity (Bouwmeester et al. 2020). However, the germination of *Orobanche cumana*, a root parasitic plant that highly specifically parasitizes sunflower, was shown to be primarily induced by STLs (Joel et al. 2011; Raupp and Spring 2013). The root exudate of sunflower contains STLs such as dehydrocostuslactone, costunolide, tomentosin, and 8-epixanthatin (Joel et al. 2011; Raupp and Spring 2013). This strongly suggest that one or more STLs can bind to one of the *O. cumana* KAI2/HTL receptors and induce KAI2/HTL signaling and thus germination.

A recent study showed that the exposure of sunflower cotyledons to blue light results in a fast accumulation (< 2 h) of 8-epixanthatin and tomentosin in the hypocotyl, which continues until reaching a maximum at 72 h (Spring et al. 2020). The other STLs, dehydrocostus lactone (which is abundantly present in roots) and costunolide, were only present in trace amounts and their concentrations did not change over time. This suggests that particularly 8-epixanthatin and tomentosin are responsible for the inhibition of hypocotyl elongation. The four STLs are also present in dry sunflower seeds, although in very low concentration. Considering the role of KAI2 signaling in germination, they may therefore also be involved in (sunflower) seed germination.

Based on all the above, we postulate that the STLs, 8-epixanthatin and tomentosin, in sunflower are the main ligands of HaKAI2s. To test our hypothesis, we identified two *KAI2* homologs from the annotated genome sequence of *H. annuus*, and performed homology modeling using YASARA version 19.12.14 (Fig. 1a, b). The percentage identity of HaKAI2a and HAKAI2b is 86% (233 out of 279 amino acid residues are identical). The 3D structures of the four STLs were obtained from the PubChem Compound library as sdf files. For molecular docking, we used the Autodock Vina algorithm integrated in YASARA using the protein models with the highest *Z* Score.

**Fig. 1:**
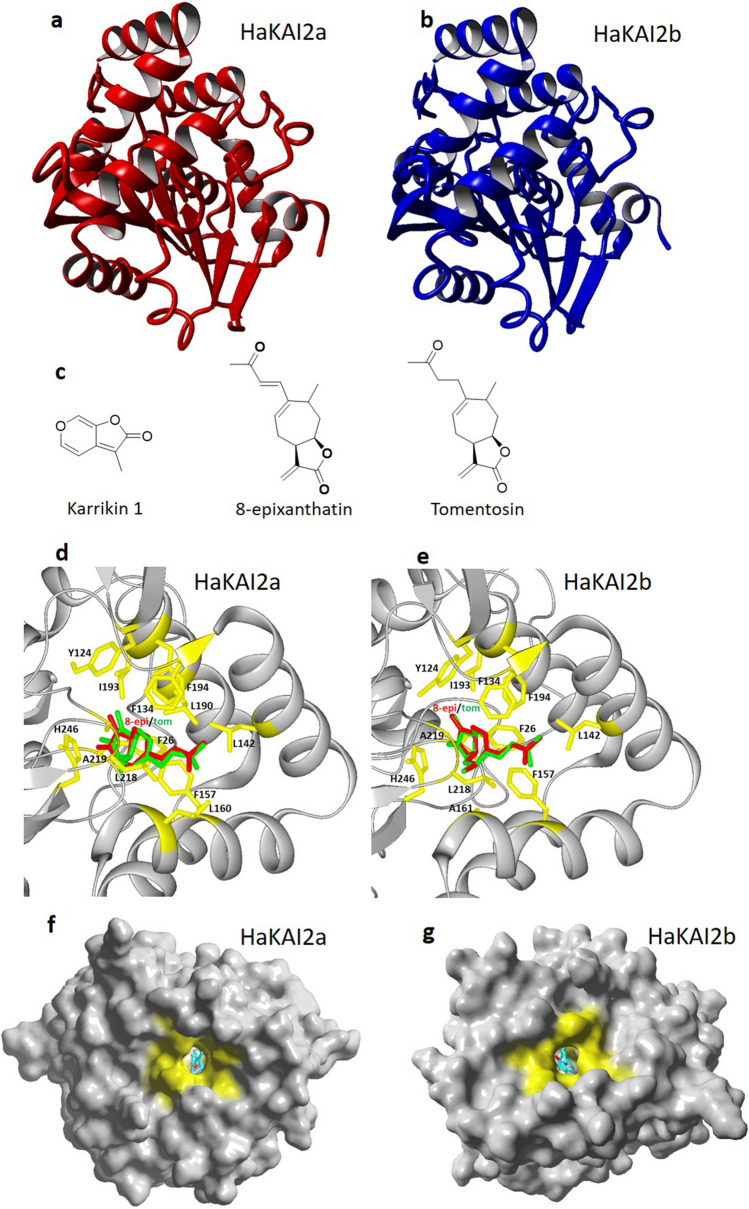
Homology models of HaKAI2s and their interaction with STLs. **a** and **b** Homology model of HaKAI2a (in red) and HaKAI2b (in blue). The overall *Z* score (comprising dihedral, and 1D packing and 3D packing *Z* scores) for the HAKAI2a and HaKAI2b models is − 0.07 and − 0.09, respectively. **c** Chemical structures of karrikin1, 8-epixanthatin, and tomentosin. **d** and **e** The hydrophobic-binding pocket of HaKAI2a (**d**) and HaKAI2b (**e**) accommodates 8-epixanthatin (8-epi, red) and tomentosin (tom, green). The amino acid residues involved in the interaction with 8-epixanthatin and tomentosin are shown in yellow. **f** and **g** 8-Epixanthatin (**f**) and tomentosin (**g**) fit into the hydrophobic pockets of HaKAI2a and HaKAI2b. The hydrophobic amino acid residues in the pockets are marked in yellow

Molecular docking showed that 8-epixanthatin and tomentosin fit into the hydrophobic-binding pockets of HalKAI2a and b (Fig. 1c, f) with high binding affinity (Kd of 3.2 and 2.7 µM for 8-epixanthatin and 6.0 and 6.2 µM for tomentosin for HaKAI2a and b, respectively). The hydrophobic amino acid residues involved in the binding of the STLs in the pocket are depicted in Fig. 1c, d. Dehydrocostus lactone and costunolide do not fit properly in the hydrophobic pockets of the modeled receptors. As expected, also KARs (KAR1 and 2) bind the hydrophobic pockets of HalKAI2a, but with *c.* 25- to 50-fold lower affinity than the STLs (Kd of 165 µM for KAR1 and 195 µM for KAR2). Surprisingly, Arabidopsis KAI2 also strongly binds 8-epixanthatin and tomentosin with estimated Kd values of about 1 and 2 µM, respectively, compared with a Kd value for KAR1 of 57 µM, a significantly lower affinity than for the STLs. The latter value matches well with the experimentally obtained Kd of AtKAI2 for KAR1, which ranges from 4.6 to 148 µM (Sun et al. 2020).

KAI2 has the catalytic triad residues, Ser95-Asp217-His246, capable of hydrolyzing butanolide substrates (Nakamura et al. 2013; De Saint Germain et al. 2016; Yao et al. 2016). However, KAI2 hydrolysis activity for KARs has not been reported (Xu et al. 2016). Indeed, it is under discussion whether hydrolysis is required for signal transduction (Zhao et al. 2013; Shabek et al. 2018; Seto et al. 2019; Yao and Waters 2020). Thus, KL may be a compound or a group of compounds that cannot undergo hydrolysis and share structural similarity with KARs. Our results suggest that STLs may be a member of this group. Sesquiterpenes have been detected in all vascular plants, and in many other organisms such as insects and fungi. Also Arabidopsis produces a large variety of sesquiterpenes (Tholl et al. 2005; Ro et al. 2006). Oxidized derivatives of these sesquiterpenes could be potential candidates for KL, although, to our knowledge, STLs have not yet been reported in Arabidopsis.

With this study, we provide candidate ligands, consisting of known endogenous plant metabolites, that can bind to KAI2 with an affinity that is much higher than for the only known exogenous ligand KAR. This finding opens up a new path to elucidate the mysterious ligand of KAI2, an important open question in plant biology with large potential applications in agriculture. Our work supports the hypothesis of others that the STLs are a plant growth regulator involved in growth and development of sunflower. We show that this effect is likely due to the interaction with KAI2. Future experimental data should validate our computational work. Studies into metabolites, possibly sesquiterpenoid, with similar lactone ring structure in Arabidopsis and other flowering plants should further solve the long-standing enigma of the elusive KL in the rest of the plant kingdom.

## Author contribution statement

H.B. and M.R. designed the project, M.R. did the modelling, and M.R. and H.B. wrote the article.
